# Skin wetness detection thresholds and wetness magnitude estimations of the human index fingerpad and their modulation by moisture temperature

**DOI:** 10.1152/jn.00538.2020

**Published:** 2021-04-07

**Authors:** Charlotte Merrick, Rodrigo Rosati, Davide Filingeri

**Affiliations:** ^1^THERMOSENSELAB, School of Design and Creative Arts, Loughborough University, Loughborough, United Kingdom; ^2^Procter and Gamble Service GmbH, Frankfurt am Taunus, Germany; ^3^THERMOSENSELAB, Skin Health Research Group, School of Health Science, University of Southampton, Southampton, United Kingdom

**Keywords:** hygrosensation, psychophysics, skin, thermoreceptors, wetness

## Abstract

Humans often experience wet stimuli using their hands, yet we know little on how sensitive our fingers are to wetness and the mechanisms underlying this sensory function. We therefore aimed to quantify the minimum amount of water required to detect wetness on the human index fingerpad, the wetness detection threshold, and assess its modulation by temperature. Eight blinded participants (24.0 ± 5.2 yr; 23.3 ± 3.5 body mass index) used their index fingerpad to statically touch stimuli varying in volume (0, 10, 20, 30, 40, or 50 mL) and temperature (25, 29, 33, or 37°C). During and after contact, participants rated wetness and thermal sensations using a modified yes/no task and a visual analog scale. The wetness detection threshold at a moisture temperature akin to human skin (33°C) was 24.7 ± 3.48 mL. This threshold shifted depending on moisture temperature (*R* = 0.746), with cooler temperatures reducing (18.7 ± 3.94 mL at 29°C) and warmer temperatures increasing (27.0 ± 3.04 mL at 37°C) thresholds. When normalized over contact area, the wetness detection threshold at 33°C corresponded to 1.926 × 10^−4^ mL·mm^−2^ [95% confidence interval (CI): 1.873 × 10^−4^, 1.979 × 10^−4^ mL·mm^−2^]. Threshold differences were reflected by magnitude estimation data, which were analyzed using linear regression to show that both volume and moisture temperature can predict magnitude estimations of wetness (*R* = 0.949; *R* = 0.179). Our results indicate high sensitivity to wetness in the human index fingerpad, which can be modulated by moisture temperature. These findings are relevant for the design of products with wetness management properties.

**NEW & NOTEWORTHY** The perception of wetness is a fundamental sensory experience which underpins many aspects of life, from homeostasis to enjoyable experiences. Although previous research has highlighted the importance of cold sensations in human wetness perception, the maximum sensitivity of our wetness sensing system remains to be established. This research presents a novel methodology, which for the first time, has quantified the high sensitivity of the human index fingerpad to wetness and its modulation by moisture temperature.

## Graphical abstract

**Figure:**
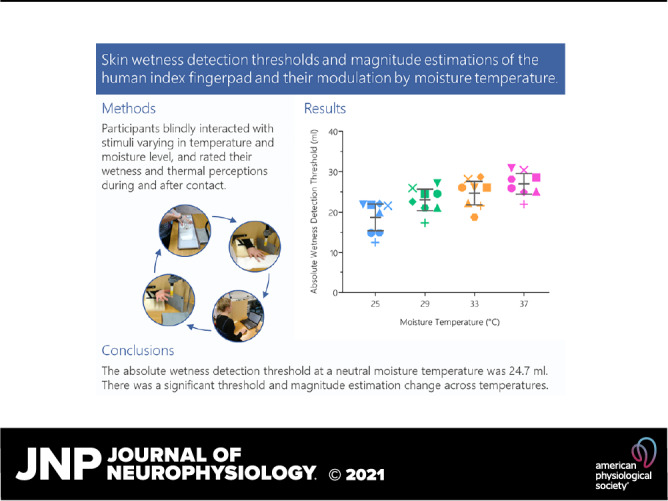

## INTRODUCTION 

Wetness perceptions are experienced by humans on a daily basis, such as holding a drink or touching a damp cloth. However, human skin has not yet been found to contain hygroreceptors, a specific receptor for wetness in the skin (1). Instead, our brains must form a comprehensive picture of the outside world by integrating a range of external stimuli such as tactile and thermal stimuli (2). The ability to sense wetness has been a critical factor in the evolution of humans and is an intrinsic component of both survival and comfort (3). This applies on a fundamental level surrounding wider homeostatic processes such as thermoregulation (4) and maintaining ion concentrations in fluid systems as part of body water balance (5). There is also a behavioral and learning element, such as being able to sense and react to environmental conditions including rain and humidity fluctuations (6) or being able to carry out specific interactions including object discrimination and precision grip (7).

Although there is a clear importance for the role of wetness perception in humans, there is not yet a conclusive model that integrates the range of senses underpinning it. Current research indicates that thermal stimuli have a significant role in wetness perception, with cold sensations being one of the main sensory drivers (8). Although cold thermal influences are considered to be the dominant modality of these potential contributors to wetness perception, little quantitative research exists surrounding the wetness detection threshold of the human index fingerpad and the effect that moisture temperature has on this. This is highly relevant, as using hands to interact with the environment is a primary exploratory action and forms the foundation of many sensorial and learning experiences for infants and adults alike (9).

Those studies that do attempt to quantify influences on wetness perception are not truly comparable due to highly variable methodological and analytical approaches. For example, Sweeney and Branson (10) reported relative detection threshold values of 4.70 × 10^−4^ mL·mm^−2^ when using polycotton on scapular hairy skin, whereas Ackerley used knitted cotton as part of a dynamic interaction to investigate the variation in wetness perception across the body, including the glabrous skin on the palm of the hand. Although no spatial effect was found across body sites, participants were able to differentiate between wetness levels and showed the lowest standard deviation in the hand (11). Although these studies had different material choices, there are other variations to be accounted for such as applied pressures and interaction duration or velocity. Furthermore, the aforementioned studies did not isolate the uppermost section of the fabric that was acting as an interface with the skin, and neither measured the specific surface area occupied by the applied liquid, instead using the full surface area of the sample in calculations regardless of liquid distribution.

On this basis, we first aim to quantify the wetness detection threshold of the human index fingerpad when interacting with wet stimuli with a temperature akin to that of skin temperature (i.e., a neutral moisture temperature), and second to detail its potential modulation by moisture temperature in terms of both threshold and magnitude estimation. We hypothesize that lower moisture temperatures will provide a wetter sensation and therefore reduce wetness detection thresholds. The outcomes will provide further evidence into an integratory model and will also be used to inform the design of superabsorbent products such as diapers, sanitary towels and incontinence pads.

## METHODS

### Participants

Eight females were recruited for the study [age 24.0 ± 5.2 yr; body mass index (BMI) 23.3 ± 3.5 kg/m^2^]. This number was established using a sample size calculation with an α value of 0.05, a β value of 0.20, and an effect size (*f*) of 0.50 based on data from pilot studies (G*Power 3.1.9.2 software; Heinrich Heine Universität, Düsseldorf, Germany). Participants were subject to an inclusion criterion, such that all were healthy nonsmoking individuals between the ages of 18 and 35 yr with a BMI below 30 kg/m^2^. Individuals were not taking any long-term medication, nor did they have any long-term somatosensory disease. All participants had a low alcohol consumption, classified as being below the recommended weekly alcohol intake.

The study design was approved by the Loughborough University Ethics Committee, and testing procedures were in accordance with the tenets of the Declaration of Helsinki. All participants were informed of the test procedures and given the opportunity to ask questions. All participants completed a health screen questionnaire and were asked to specify the dates of their most recent menstrual period. All participants gave their informed written consent before participation. Prior to the scheduled testing, participants’ body mass and height were recorded with a scale (ID1 MultiRange, Mettler, Toledo, OH) and stadiometer (HM-250P, Marsden, UK) to determine their BMI and hence confirm eligibility for the study.

### Experimental Design

The study was conducted as a single blind psychophysical experiment, such that participants were unaware of information that may bias the results. Diapers were chosen as the stimulus for investigation of thermal cues. They are composed of four layers: the outer chassis, superabsorbent inner core, acquisition layer, and finally the topsheet, which is the uppermost layer in contact with the skin. The unique liquid sequestering properties offered by diapers allows a large range of volumes to be used with their temperatures easily maintained. This is facilitated by an absorbent gelling material, a type of superabsorbent polymer that that can absorb and retain an incredibly large volume of liquid relative to its size (12). The capacity of the superabsorbent depends on the polarity and ionic concentration of the applied liquid (13). In the context of diapers this liquid is urine, which can be mimicked using saline (14). This poses no anticipated disruption to perceptual data collection, as current research indicates that wetness sensations are primarily driven by cold thermal inputs (8) and there are minimal dermatological effects of such short-term saline exposure (15).

The pilot phase of the study focused on wetness perceptions using 0.9% saline (10.8 ± 0.02 g NaCl; 1,200 ± 10 mL H_2_O) across a wide range of volumes (0 mL, 25 mL, 50 mL, 75 mL, 100 mL, and 125 mL). These were chosen to reflect the wide absorbency range of the diapers. Additionally, by including stimuli that were completely dry and stimuli wetted to the point of saturation, this gave negative and positive controls respectively. This allowed the broader estimation of the wetness detection threshold and gave associated perceptual magnitudes. These data were used to isolate and apply a more specific volume range (0 mL, 10 mL, 20 mL, 30 mL, 40 mL, and 50 mL) around the threshold in the full experimental study and hence improve resolution. In both pilot and full experiments, each participant completed four experimental sessions in which the same quantitative sensory tests were conducted using different stimulus moisture temperatures in each respective session (25°C, 29°C, 33°C, and 37°C). Herein, all information refers to the full experiment as opposed to the pilot unless otherwise stated.

Within each session the aforementioned six volumes (0 mL, 10 mL, 20 mL, 30 mL, 40 mL, and 50 mL) were used to prepare stimuli, which were presented to participants after a 20-s dwell time had elapsed to allow moisture distribution throughout the superabsorbent layer. Each volume was presented 12 times in a balanced order both within and between participants. The index finger of the nondominant hand was used to statically interact with the stimuli at a static resting pressure for 5 s. It was positioned at a shallow angle such that the nail was almost parallel to the stimulus. This stimulus was perceptually rated at initial touch and immediately after withdrawing. Both a dichotomous response method (dry/wet, cold/warm) and a 100-mm visual analog scale (very dry to very wet, cold to warm) were used for during- and postcontact evaluations.

The nondominant hand was chosen to interact with the stimulus as the dominant hand was required to fill in the scales. This is unlikely to affect perceptions as thermal and tactile sensitivity have been found to be the same on each index finger (16), and similarly nociceptors were triggered at the same intensity on each side (17). As resting pressure reflects the hand in a relaxed state, this is also unlikely to vary between dominant and nondominant sides. Resting pressure is typically 1–2 N depending on finger size and will naturally vary between individuals (18). However, the distribution of receptors across the finger is proportional to its size, and so is unlikely to have an effect at resting pressure (19). In total, each participant interacted with 72 stimuli in each session, 12 repeats of six volumes at each respective temperature. After wetness detection thresholds were established from experimental data, they were converted to values relative to surface area.

Local skin temperature (T_sk_) at the stimulus contact interface was measured throughout interactions by use of a single thermocouple (0.08 mm wire diameter, 40 gauge; 5SRTC-TT-TI-40-2M, Omega, Manchester, UK). This was affixed to the center of the index fingerpad using surgical skin tape (25-mm-width Transpore, 3 M, Loughborough, UK), ensuring the thermocouple tip was in contact with the skin but not covered by tape. Note the fragility of the thermocouples occasionally caused breakages such that thermal data were not collected for every participant. Larger, more robust thermocouples did not provide sufficient resolution and so were not used. All sessions were conducted in a thermoneutral environment (23.9 ± 0.8°C, 37% RH). During sessions participants assumed a seated position and were blinded to the experimental setup using an L-shaped obscuring screen to limit visual cues. Additionally, auditory cues including stirring and pouring of saline were systematically added every 4 min during stimulus preparation procedures to counteract any associative learning effects or bias in results. This was preferable to blocking auditory cues entirely, such as via ear defenders, as this would have interfered with the verbal commands used to direct participant interactions. Participants were allowed to take short self-governed breaks during the session and were only permitted to consume water during this time.

### Experimental Protocol

Prior to the start of each experimental session, a 0.9% saline solution (8.46 ± 0.02 g NaCl; 1,200 ± 10 mL H_2_O) was prepared to mimic the ionic composition of infant urine and therefore be absorbed onto the substrate optimally. The intended application temperatures were 25°C, 29°C, 33°C, and 37°C, with the former two being within and just above the activation range of cold receptors (20) and the latter two reflecting average skin temperature and average core temperature, respectively. To account for heat losses during sample preparation and while the dwell time elapsed, each solution temperature was maintained using a small manually controlled thermal chamber at either 25.1°C, 29.2°C, 33.4°C, or 37.7°C ± 0.1°C in the four respective sessions. These temperatures were established during initial sample classification studies using the same stimulus preparation protocols and experimental conditions as the full study, but across a wider range of moisture temperatures, with thermocouples embedded to monitor the thermal equilibration patterns toward room temperature.

Different volumes of saline solution were applied to individual stimuli to moisten them before interaction with a participant. The stimuli comprised the superabsorbent core and associated layers from the center of a diaper. This “center” was cut from the elasticated diaper chassis such that it laid flat, as opposed to curving with the body as designed. Cuts were made such that the superabsorbent core was not ruptured, and no internal material was lost or made prone to leakage. This resulted in a 115-mm × 325-mm rectangular sample. In the pilot, the applied volume of saline was either 0 mL, 25 mL, 50 mL, 75 mL, 100 mL, or 125 mL. In the full experiment, this was refined to 0 mL, 10 mL, 20 mL, 30 mL, 40 mL, and 50 mL.

**Figure 1.**
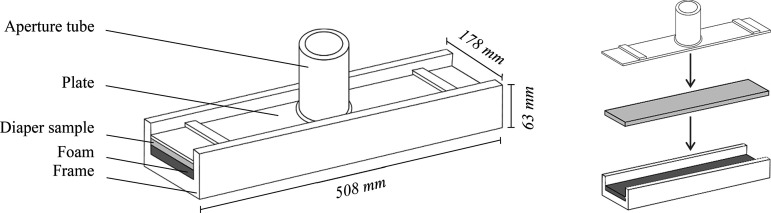
A diagram of the acquisition plate and associated assembly used to apply liquid to the diaper samples.

Each volume was applied to the substrate using a custom-made acquisition plate (Fig. 1). This was formed of a plastic frame and foam stage upon which the sample would be placed, followed by a flat plate with aperture tube above it. When the diaper was aligned correctly between the terminal markers, the tube was positioned directly above its center. The desired volume of solution was then applied via the aperture tube using a graduated plastic syringe (SS + 50ES1, Terumo, Leuven, Belgium). After the solution had been absorbed from the aperture tube, the sample was allowed to rest for a period of 20 s, termed the dwell time. This period effectively allowed the solution applied to the topsheet to be absorbed by the acquisition layers and wicked away from application area to ensure a uniform distribution.

Prior to experimental interactions, participants would undergo familiarization to ensure the correct use of psychometric scales at the start of each of the four sessions. The familiarization protocol consisted of four stimuli combinations; cold-wet, warm-wet, cold-dry, and warm-dry. Each stimulus was introduced to the participant under standard test conditions, and the corresponding response on the psychometric assessments shown. As the stimuli were combinations that demonstrated the extremes of each condition across the experimental sessions, they provided a frame of reference for the study in addition to acquainting participants with the study protocols.

Upon completion of familiarization, participants inserted their nondominant hand through an aperture in an L-shaped screen which prevented them seeing the experimental setup. The base was lined with foam to reduce conductive heat transfer and could be inverted to accommodate different hand dominances. Participants were instructed to place their index finger on to a fixed-position thermoneutral plate, which would maintain T_sk_ at 33°C, herein also termed neutral. This effectively established a baseline from which interacting with a specific stimulus would result in the same rate of heat transfer across participants, thus minimizing inter- and intraindividual variability (21). When the stimulus had been prepared as aforementioned, the participant was given the command “contact.” They would then move their hand from the thermoneutral plate and make contact with the stimulus at a static resting pressure.

Participants had previously been instructed on such commands, and the correct orientation of the finger demonstrated and practiced. Additionally, the stimulus was always positioned correctly below the finger and moved before contact when necessary. At the point of contact participants would immediately complete a digital perceptual form based on the during-contact interaction within 3 s. Both a dichotomous response method (dry/wet, cold/warm) and a 100-mm visual analog scale (very dry to very wet, cold to warm) were employed. The dichotomous response paradigm used a binary scoring system for subsequent analyses, with a “dry” response designated as 0 and a “wet” response a 1 for wetness perceptions. Similarly, a “cold” response was coded as 0 and a “warm” response as 1 for thermal assessments.

After a contact period of 3 s, the participant was prompted to remove their finger from the stimulus using the command “lift.” Postcontact perceptual assessments identical to those used in the during-contact interaction were be completed, again within 3 s. Participants indicated completion using the word “done,” at which point the stimulus would be removed and replaced with a cotton towel. The participant was then instructed “dry” and would statically press their index finger on to a dry cotton towel to collect residual water for 5 s. This was repeated for all stimuli regardless of wetness to prevent any learning effect or bias. In between this stimulus and the next, the index finger was returned to the thermoneutral plate to maintain T_sk_ at 33°C. The period in which the finger was on the thermoneutral plate also served as a nervous refractory period lasting a minimum of 20 s, during which time the next stimulus was prepared before repeating the protocol.

### Threshold Determination

Individual thresholds were determined using a modified dichotomous response method. There are two methods typically employed in threshold determination, which are considered to be equally effective in different physiological measures. A classic two-alternative forced choice method allows participants to choose which of two stimuli correspond best to a single descriptor, whereas the associated yes/no task involves only a single stimulus to which either a positive or negative response must be assigned (22, 23). The current methodology incorporates principles from both, allowing participants to classify a single stimulus from two opposing descriptors (dry/wet, cold/warm).

**Figure 2.**
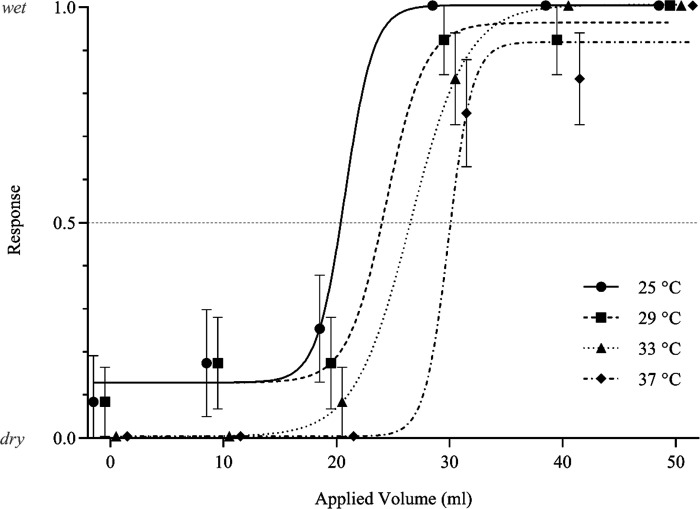
A/n example of the during-contact wetness detection thresholds of a single female participant (*n* participants =* *1), as determined via the dichotomous response method using sigmoidal curves. Note the equal numbers of data points above and below threshold. Each data point represents x̅ ± 95% CIs wetness response from 12 repeat stimuli presented to a participant at a specific applied volume and temperature. CIs, confidence intervals; x̅, mean.

Coding the dichotomous responses as 0 or 1 allowed an average response ratio to be generated at each applied volume. These ratios were plotted across all applied volumes and fitted with a logistic sigmoidal curve, an s-shaped fit typically used to establish thresholds. The point at which the curve crossed 0.5 was decided as the detection threshold, on the basis that approximately half of values would feel dry, subceeding the threshold, and half would feel wet, exceeding it. However, sigmoidal curves generated across the test temperatures in the pilot studies indicated that the wetness detection thresholds for all temperatures lay between 15 mL and 35 mL, which falls at the lower end of the range of tested volumes of 0 mL–125 mL. Beyond this the curves peaked and plateaued, resulting in a poor overall fit. Despite this, there was still a notable difference in overall perception across temperatures, with lower temperatures being associated with lower wetness detection thresholds. These data allowed the secondary volume range of 0 mL–50 mL to be established, centering the range around the proposed 0.5 threshold by providing a roughly equal quantity of stimuli below and above threshold (Fig. 2), hence providing a balanced design and validating the use of the chosen threshold value (8). Additionally, this informed setup promotes a superior fit, allowing a higher resolution surrounding the detection threshold to be achieved in the full experiment, and also reduces anticipatory bias that could be expected by participation in a “wetness perception” study, which inherently implies the presence of moisture in stimuli.

### Relative Threshold Determination

Following the collection of perceptual and physiological participant data, the established absolute wetness detection threshold values were converted into relative values, which relied on several principles. The first of these was the isolation of the topsheet of the diaper, which is the uppermost layer in contact with the participant’s finger and therefore the acting interface between skin and moisture. Leading on from this, the level of moisture contained within the topsheet itself was critical. By establishing the surface area which it occupied and the corresponding change in weight of the topsheet, the relative water retention could be calculated within a given area.

**Figure 3.**
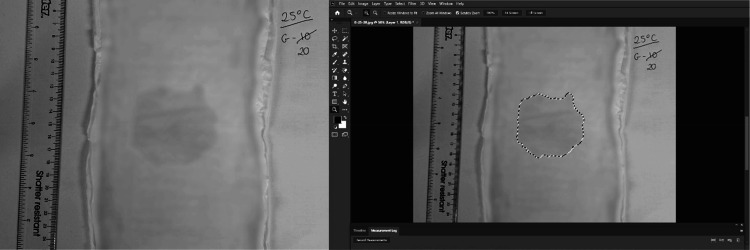
The original photo of the sample (*left*) and part of the surface area measurement process (*right*).

This was achieved by first isolating the aforementioned dry topsheet of the stimulus and weighing it. Subsequently a blue dye was added to the saline solution and applied at all experimental volumes under test conditions. However, instead of a participant interaction with the sample, the topsheet was immediately removed and weighed after the 20-s dwell time had elapsed. Having previously weighed the dry topsheet, the difference in weight between this and the wetted topsheet could be used to infer saline content, with 1 mL weighing ∼1 g. Using a top-down tripod clamp, a photograph was then taken of the topsheet with the inclusion of a scale such that the exact surface area, as indicated by the blue dye, could later be established using digital imaging software (Photoshop 2017.0.0, Adobe, San Jose, CA; Fig. 3).

The relationship between applied volume and surface area within the tested range was directly proportional. The absolute saline content and corresponding surface area could then be used to calculate the relative surface wetness of the sample topsheet (*Eq. 1*). Calculation of surface wetness as a function of liquid content in a given area can be done with the following equation:

(*1*) $${\text{Surface wetness (mL·mm}}^{-\text{2}}\text{) = }\frac{\text{saline content (mL)}}{{\text{surface area (mm}}^{\text{2}}\text{)}}$$

**Figure 4.**
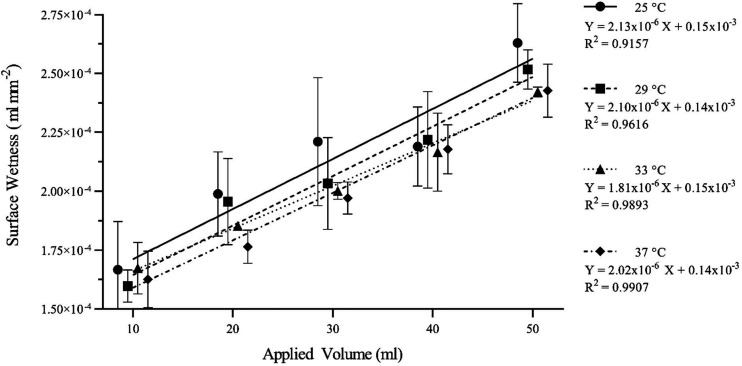
The x̅ ± 95% CIs (*n * replicates =* *9) surface wetness in the topsheet plotted across the range of applied volumes for each temperature condition, with associated linear regression equations. CIs, confidence intervals; *X*, applied volume; *Y*, calibrated relative detection threshold; x̅, mean.

By plotting the established surface wetness of the topsheet across the range of applied volumes for each temperature condition, regression equations could be generated (Fig. 4). From these, a standardizing equation for surface wetness could be established (*Eq. 2*). In this regression equation, the X value corresponds to an applied volume. As the absolute wetness detection threshold was calculated in terms of an applied volume it can be inputted as *X*. This generates a *Y* value for surface wetness in the topsheets, which can be considered as the calibrated relative detection threshold. Calculation of surface wetness in the topsheet using a previously generated linear regression equation can be expressed as follows:

(*2*) $${\text{Relative surface wetness (mL·mm}}^{-\text{2}}\text{) = gradient }\text{× absolute threshold (mL) + intercept}$$

### Statistical Analyses

In this study, the independent variables were the temperature of the stimuli (25°C, 29°C, 33°C, 37°C) and the applied volume (0 mL, 10 mL, 20 mL, 30 mL, 40 mL, 50 mL). The dependent variables were absolute wetness detection thresholds, wetness perception (mm), thermal sensation (mm), and fingerpad T_sk_ (°C). Absolute wetness detection thresholds are specified in mL, and relative wetness detection thresholds are specified in mL·mm^−2^. All data were tested for normality of distribution and homogeneity of variances using Shapiro-Wilk and Levene’s tests, respectively. In cases where the assumptions of these tests were violated, parametric means-based tests were nonetheless applied as they best fit the required analyses of the datasets. All statistical data reported in text are means (x̅) ± standard deviation (SD), with means and 95% confidence intervals (95% CIs) given in figures unless otherwise stated; α = 0.05. All statistical analyses were conducted using SPSS (Statistical Package for Social Sciences, V. 24.0.0.2, IBM, Chicago, IL) and graphical representations of data produced using GraphPad Prism (GraphPad Prism, V. 8.3.0, GraphPad Software, La Jolla, CA).

During contact, absolute wetness detection thresholds were established using dichotomous data, by plotting mean binary perceptual scores from each participant against applied volume. For example, 5 dry responses and 7 wet responses would generate a value of 0.42. This was analyzed as part of a logistic sigmoidal curve fit using a lapse rate of zero, assumed given the responses from pilot studies. The point at which the response rate exceeds 0.5 (50%) indicates that the data are no longer due to chance, and so can be considered threshold. Threshold values were established for each of the eight participants at each of the test temperatures and converted into relative values. The mean of these individual thresholds was subsequently calculated to give an overall mean relative wetness detection threshold for each test temperature. To investigate the influence of applied temperature on during-contact absolute wetness detection thresholds, a parametric one-way ANOVA was conducted. The results were further analyzed using post hoc Tukey tests.

Magnitude estimation data were used to assess the influence of applied volume and applied temperature during- and postcontact wetness perception. These were processed using linear regression in conjunction with a two-way ANOVA. The difference between during- and postcontact wetness perceptions was then compared using paired *t* tests. Further to this, magnitude estimation data were also used to assess the influence of applied volume and applied moisture temperature on during- and postcontact thermal perceptions. The data were initially analyzed using a two-way ANOVA with post hoc pairwise Tukey tests to establish whether both during- and postcontact thermal perceptions significantly differed at different applied volumes and moisture temperatures, respectively. A linear regression analysis was then conducted to assess the overall contribution of applied volume and applied moisture temperature on during- and postcontact thermal perception. The postcontact thermal perceptual data were then compared with during contact, and the relationship analyzed using a series of paired *t* tests.

T_sk_ data from thermocouples were plotted to validate applied temperatures and time intervals. Additionally, the average T_sk_ at each temperature was compared during and postcontact with a paired *t* test. Linear regression analyses were used to assess the relationship between during- and postcontact T_sk_ in relation to during- and postcontact thermal perceptions. Finally, a linear regression analysis was conducted to assess the independent and interactive influence of applied volume and moisture temperature on the magnitude estimation of wetness perception.

## RESULTS

### Wetness Detection Thresholds

**Figure 5.**
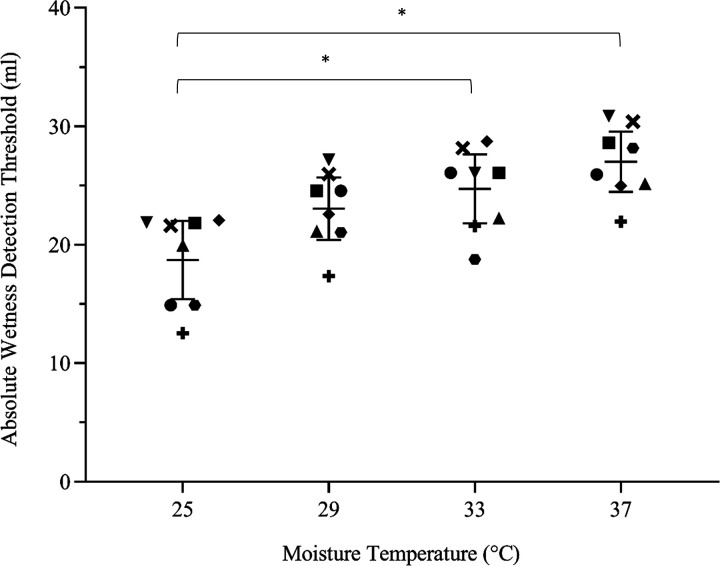
The x̅ ± 95% CIs (*n* participants =* *8) during-contact absolute wetness detection thresholds of the human index fingerpad. Data points represent individual thresholds from eight female participants across the four moisture temperatures, with each individual indicated by a different shape. *Significant moisture temperature pairings following statistical analysis with a one-way ANOVA and post hoc Tukey tests. CIs, confidence intervals; x̅, mean.

Use of a dichotomous response method enabled the determination of the absolute wetness detection threshold of the human index fingerpad at the moisture temperature resembling human skin temperature (i.e., 33°C) to be 24.7 ± 3.48 mL. This threshold varied according to applied moisture temperature. Temperatures below 33°C resulted in lower wetness detection thresholds, with 25°C and 29°C having absolute thresholds of 18.7 ± 3.94 mL and 23.0 ± 3.17 mL, respectively. At 37°C, the threshold increased to 27.0 ± 3.04 mL (Fig. 5). This gave a total wetness detection threshold range of 8.3 mL between lowest and highest temperatures. This relationship between applied temperatures and wetness detection thresholds was statistically significant (*F*_3,21_ = 8.79; *P* = 0.002), with post hoc analyses indicating a statistical difference between 25°C and 33°C pairings (Δx̅ = −6.00; CIs = −12.0, −0.0160), as well as 25°C and 37°C pairings (Δx̅ = −8.29; CIs = −15.5, −1.09).

Each temperature had a corresponding calibration equation which allowed expression of absolute detection thresholds (mL) as relative values (mL·mm^−2^) according to the moisture content retained and relative surface area occupied in the uppermost layer of the stimulus. Table 1 presents the mean masses of the uppermost layer of the stimulus before and after the addition of moisture, with corresponding mean surface area and mean surface wetness.

**Table 1.** Mass of the uppermost layer of the stimulus before and after loading with respective surface characteristics at each applied moisture level

Applied Moisture, mL	Mass before Load, g	Mass after Load, g	Change in Mass, g	Mean Surface Area, mm^2^	Mean Surface Wetness, mL·mm^−2^
10	1.52	1.82	0.296	1,810	1.64 × 10^−4^
20	1.49	2.09	0.606	3,230	1.88 × 10^−4^
30	1.62	2.45	0.828	4,040	2.05 × 10^−4^
40	1.53	2.97	1.44	6,580	2.19 × 10^−4^
50	1.45	3.47	2.01	8,060	2.50 × 10^−4^

Table 2 presents absolute and relative wetness detection thresholds at each moisture temperature. The relationship between applied moisture temperatures and wetness detection thresholds was analyzed in absolute terms, as larger temperatures are more stable. Additionally, any uncertainties in the surface area of topsheet liquid content used to determine relative thresholds will be magnified in this form of analysis.

**Table 2.** Absolute and relative wetness detection thresholds of eight female participants with mean and standard deviation

Applied Temperature, °C	Absolute Wetness Detection Threshold, mL	Relative Wetness Detection Threshold, mL·mm^−2^
25	18.7 ± 3.94	1.896 × 10^−4^ ± 8.39 × 10^−5^
29	23.0 ± 3.17	1.918 × 10^−4^ ± 6.67 × 10^−5^
33	24.7 ± 3.48	1.926 × 10^−4^ ± 6.30 × 10^−5^
37	27.0 ± 3.04	1.933 × 10^−4^ ± 6.14 × 10^−5^

Values are mean (x̅) ± SD.

### Magnitude Estimation of Wetness Perception

**Figure 6.**
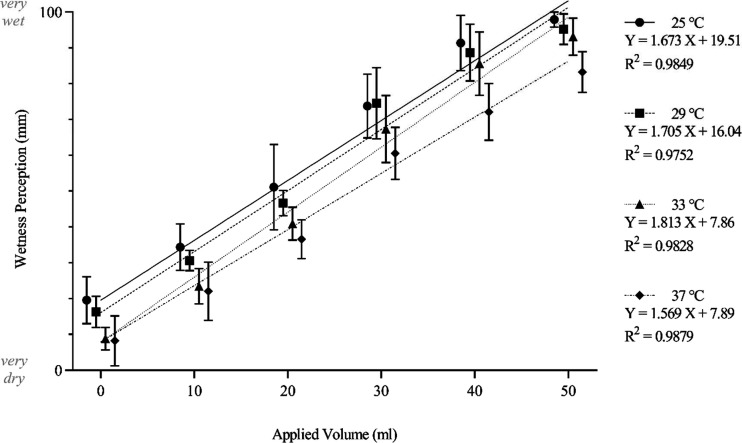
The x̅ ± 95% CIs (*n* participants =* *8) during-contact wetness perception ratings of stimuli presented to eight female participants at a range of applied volume and temperature combinations, fitted using linear regression. CIs, confidence intervals; x̅, mean; *X*, applied volume;* Y*, calibrated relative detection threshold.

The effects of applied volume and applied temperature on during-contact wetness perception were reflected in magnitude estimation data. Applied volume had a proportional relationship with perceived wetness perception, such that greater volumes resulted in higher wetness perceptions. Conversely, applied temperature was inversely proportional to wetness perception such that lower temperatures resulted in greater wetness perceptions (Fig. 6). The data showed a statistically significant difference between perceived wetness at different applied volumes (*F*_5,35 _= 3,010; *P* < 0.001) and applied temperatures (*F*_3,21_ = 177; *P* < 0.001). Applied volume accounts for 84.0% of variance in during-contact wetness perceptions, whereas applied temperature only accounts for 2.96%. Even so, there is a significant interaction between the two factors such that they act synergistically to produce a compounding effect (*F*_15,105_ = 3.73; *P* < 0.001), which accounts for 0.31% of variance.

The relationship between magnitude of wetness perception and applied volume is reflected by linear regression lines at different temperatures. The linear regression lines appear shifted according to temperature, with 25°C resulting in the greatest overall wetness perceptions and 37°C resulting in the lowest. This pattern is similar in *y*-intercept values, which indicates perceived wetness in dry conditions, with 33°C and 37°C predicting a similarly low perceived wetness and the cooler values of 29°C and 25°C resulting in progressively larger wetness perceptions. At the other end of the scale, it can be noted that the data points across all temperatures become clustered slightly below the linear regression line at the applied volume of 50 mL. This is likely a result of the upper extremity of the scale being approached, at which point participants typically become more conservative and show a reduced accuracy (24). It is also possible that participants were experiencing a ceiling effect, but the potential of this was minimized by providing reference stimuli during familiarization.

The effects of applied volume and applied temperature on postcontact wetness detection were reflected in magnitude estimation data. As with during-contact wetness perception, applied volume had a proportional relationship with perceived wetness perception, whereas applied temperature was inversely proportional to wetness perception. There was a statistically significant difference between perceived wetness at different applied volumes (*F*_5,35_ = 1,430; *P* < 0.001) and applied temperatures (*F*_3,21_ = 172; *P* < 0.001). Applied volume accounts for 71.3% of variance in postcontact wetness perceptions, whereas applied temperature accounts for 5.16%. There is a small interaction between the two factors such that they act synergistically to produce a compounding effect (*F*_15,105 _= 5.63; *P* < 0.001), which accounts for 0.84% of variance in wetness perception.

Wetness perceptions varied between during- and postcontact interactions at the different applied moisture temperatures, with stimuli being perceived as drier postcontact by an average of 3.3 mm (CIs = 2.5, 4.2), 0.4 mm (CIs = −0.4, 1.3), 1.8 mm (CIs = 0.5, 3.1), and 6.7 mm (CIs = 5.1, 8.3) at 25°C, 29°C, 33°C, and 37°C, respectively. The overall decrease in wetness perception postcontact was found to be significant at three of the four tested temperatures, the exception being 29°C (25°C, t_7_ = 7.99, *P* < 0.001; 29°C, t_7_ = 0.919, *P* = 0.359; 33°C, t_7_ =2.77, *P* < 0.006; 37°C, t_7_ = 8.09, *P* < 0.001).

### Magnitude Estimation of Thermal Perception

The effect of applied volume and applied moisture temperature on during-contact thermal perception was highlighted in magnitude estimation data. Thermal perceptions shared a positive relationship with moisture temperatures and significantly differed across applied range (*F*_3,21 _= 4,960, *P* < 0.001). Additionally, all applied moisture temperature pairings were significantly different (*P* < 0.001). Thermal perceptions were also affected by different applied volumes (*F*_5,35_ = 9.15, *P* < 0.001), such that greater volumes typically resulted in a greater magnitude of thermal perception. For example, warm stimuli were perceived to be warmer at larger volumes, and cool stimuli were perceived as cooler at larger volumes. Overall, moisture temperature was accountable for 84.0% of variance in thermal perceptions, and applied volume accounted for 0.26% of variance. Finally, there was a significant interaction between variables (*F*_15,105_ = 33.8, *P* < 0.001), with 2.86% of variance in thermal perception due to the collective influence of applied volume and temperature. This was reflected in the associated linear regression plots, with increases in the magnitude of applied volume resulting in a greater deviation of during-contact thermal perceptions from neutrality. The exception was at the neutral skin temperature of 33°C, at which thermal perception was relatively stable regardless of applied volume.

**Figure 7.**
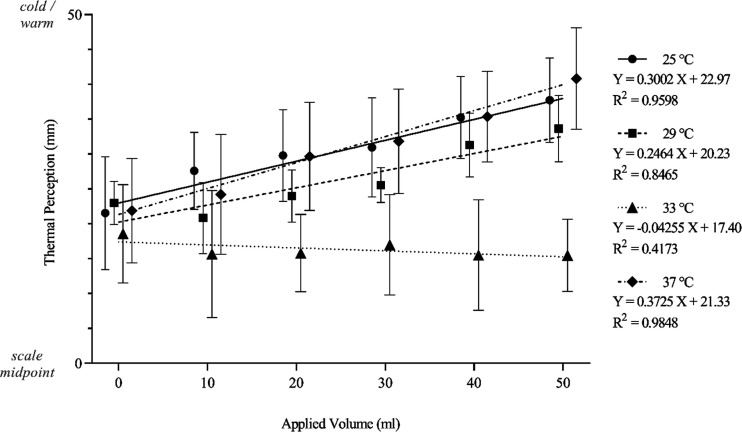
The x̅ ± 95% CIs (*n* participants =* *8) during-contact thermal perception ratings of stimuli presented to eight female participants at a range of applied volume and temperature combinations, fitted with linear regression. Ratings are expressed as a deviation from VAS midpoints, which were unmarked, to the cold and warm extremes to allow magnitude comparison without directionality. CIs, confidence intervals; VAS, visual analog scale; x̅, mean; *X*, applied volume; *Y*, calibrated relative detection threshold.

The effect of applied volume can be further investigated by expressing all thermal perception data as a deviation from the midpoint of the scale, ensuring that magnitude changes are not negated because of their opposing directions (Fig. 7). In this case, the relative effect of moisture temperature decreases to 21.7%, and the applied volume increases to account for 10.1% of variance, and finally the effect of and interaction increases to 5.8%.

The effect of applied volume and applied moisture temperature was also significant on postcontact thermal perceptions, which were significantly different at different applied volumes (*F*_5,35_ = 23.5, *P* < 0.001) and applied moisture temperatures (*F*_3,21_ = 3,040, *P* < 0.001). Linear regression analyses showed that both applied volume and moisture temperature shared a positive relationship with during-contact thermal perception. Applied volume accounted for 0.99% of variance and moisture temperature 77.0%. Additionally, there was a significant interaction between variables (*F*_15,105_ = 21.1, *P* < 0.001), with 2.68% of variance in thermal perception due to the collective influence of applied volume and temperature.

Thermal perceptions also varied in postcontact interactions, with overall differences between during-contact and postcontact found to be significant at all temperatures (25°C, t_7_ = −5.79, *P* < 0.001; 29°C, t_7_ = −11.4, *P* < 0.001; 33°C, t_7_ = 9.26, *P* < 0.001; 37°C, t_7_ = 13.6, *P* < 0.001). In postcontact interactions, perceptions tended back toward neutrality once the finger was lifted. The difference in during- and postcontact perception was proportional to the difference of the applied temperature from neutrality, with changes of −2.0 mm (CIs = −2.7, −1.3), −4.3 mm (CIs = −5.1, −3.6), 3.7 mm (CIs = 2.9, 4.5), and 7.3 mm (CIs = 6.3, 8.4) at 25°C, 29°C, 33°C, and 37°C, respectively.

### Thermocouple Data

**Figure 8.**
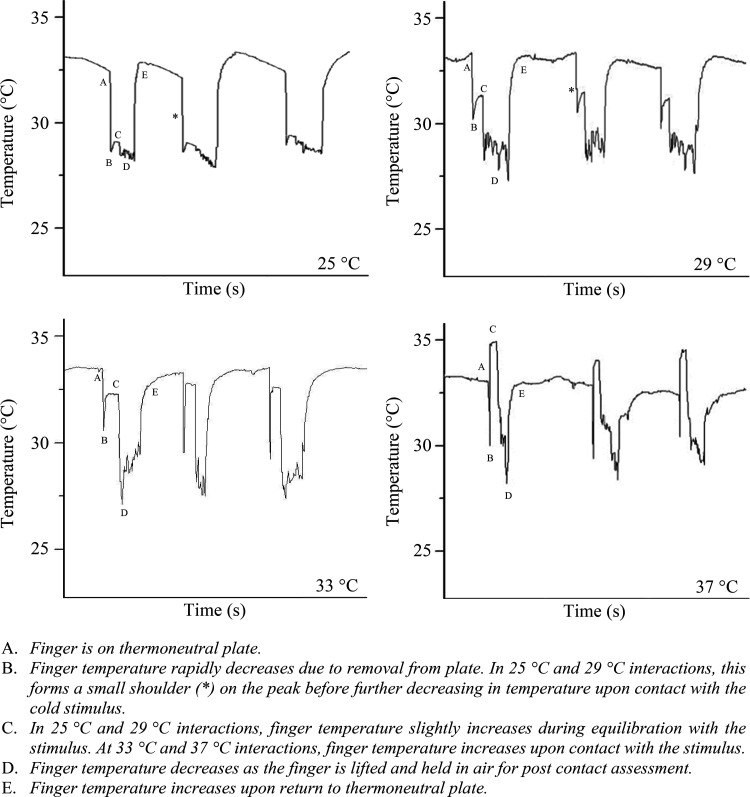
Example thermal profiles generated with thermocouples, showing the changes in skin temperature on the index fingerpad during three interactions at each of the different test temperatures (25°C, 29°C, 33°C, and 37°C). Key interaction stages labeled *A*–*E*.

The interactions between participant and stimuli made distinctive thermal traces at each applied temperature (Fig. 8). The mean T_sk_ upon transient contact with stimuli was 29.7 ± 1.14°C, 32.3 ± 0.84°C, 33.0 ± 0.66°C, and 34.8 ± 0.41°C at 25°C, 29°C, 33°C, and 37°C, respectively. The mean postcontact T_sk_, measured immediately after the finger was removed from the stimulus, was 29.0 ± 1.26°C, 32.2 ± 0.45°C, 30.7 ± 1.55°C, and 30.3 ± 0.65°C, respectively. Mean temperature differences between during- and postcontact T_sk_ were found to be significant at three of the four applied temperatures, the exception being 29°C (25°C, T_4_ = 5.63, *P* < 0.001; 29°C, T_2_ = 0.713, *P* = 0.491; 33°C, T_5_ = 12.6, *P* < 0.001; 37°C, T_3_ = 43.6, *P* < 0.001).

**Figure 9.**
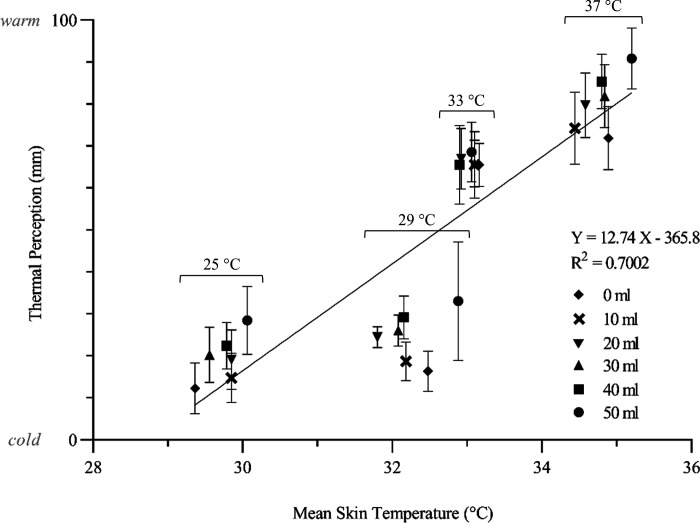
The x̅ ± 95% CIs (*n* participants =* *8) thermal perceptions of stimuli presented to eight female participants and the corresponding average skin temperature at the time of interaction, fitted with linear regression. Each data point represents one of the six applied volumes, as indicated with different symbols, and is grouped according to applied moisture temperature. CIs, confidence intervals; x̅, mean; *X*, applied volume; *Y*, calibrated relative detection threshold.

The average T_sk_ upon transient contact with the stimuli shared a positive relationship with during-contact thermal perceptions (Fig. 9), which was statistically significant (Β = 12.7, *F*_1,4_ = 444, *P* < 0.001). Average T_sk_ and thermal perception postcontact were positively correlated; however, this relationship was not significant (Β = 3.06, *F*_1,4_ = 3.75, *P* = 0.580).

**Figure 10.**
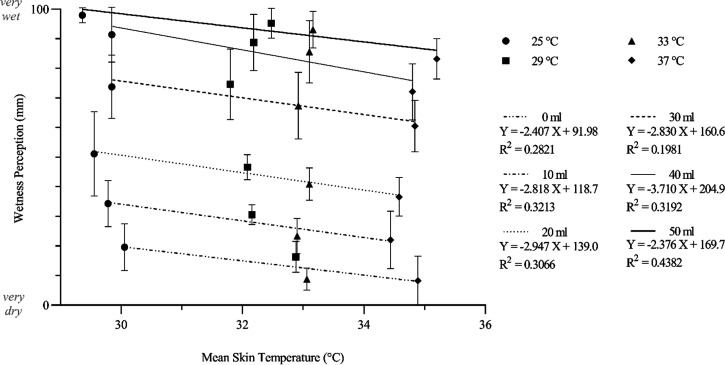
The x̅ ± 95% CIs (*n* participants =* *8) wetness perceptions of stimuli presented to eight female participants and the corresponding average skin temperature at the time of interaction, fitted with linear regression. Data points grouped according to the six applied volumes and four moisture application temperatures. CIs, confidence intervals; x̅, mean; *X*, applied volume; *Y*, calibrated relative detection threshold.

When T_sk_ was associated with wetness perception during-contact, similar trends could be identified in terms of responses at 25°C and 29°C sharing similar perceptual magnitudes despite changes in T_sk_ (Fig. 10). The aforementioned influence of applied volume can also be highlighted in relation to T_sk_.

### Multiple Regression Analysis

When the independent variables, applied volume and applied moisture temperature, are integrated into a linear regression 85.5% of variance in wetness perception during stimuli contact could be accounted for. This can be attributed to both applied volume and applied moisture temperature, which can be used to generate an equation to accurately predict perceived wetness during-contact with a stimulus, expressed using a 100-mm visual analog scale (*Eq. 3*). Linear regression model for the prediction of wetness perception based on volume and temperature is as follows.

(*3*) Perceived  wetness (mm) = 27.9 + [1.69 × applied volume (mL)] − [4.82 × applied temperature (°C)]

## DISCUSSION

### Wetness Detection Thresholds

Use of a dichotomous response method enabled the determination of the relative wetness detection threshold of the human index finger at a moisture temperature akin to human T_sk_ (33°C). Application of moisture at different temperatures modulated the threshold such that it was lower at cooler temperatures and higher at warmer temperatures, effectively showing that a greater quantity of moisture is required to illicit the sensation of wetness as temperature increases. From a physiological perspective, this implies that humans are more sensitive to wetness at cooler moisture temperatures. In this case wetness was experienced at higher temperatures in addition to those below skin temperature, albeit at a lower intensity. This shows that there must be other factors involved in wetness perception and supports previous mechanistic work. For example, cold cues have been associated with wetness in the absence of physical wetness, when using a nerve block (25) and in the absence of physical skin cooling (26). As visual and auditory cues were removed or controlled in the present study, this brings a focus back on to the roles of haptics. Although interactions were static in this case, there is potential for characteristics such as adhesive forces and textural changes to play a role in wetness perception, which requires further investigation.

### Magnitude Estimation of Wetness Perception

In relation to magnitude estimation data, similar trends were seen in terms of greater volumes being perceived as wetter. Additionally, the effect of applied temperature on wetness perception seems similar to that of the dichotomous response method, with wetness sensation being inversely proportional to applied moisture temperature. Although dichotomous response and visual analog scale methods show that the magnitude of difference in wetness perceptions is proportional to the magnitude of difference in volume, the trend is more prominent in the latter method. This is perhaps because of the higher resolution of visual analog scales, which effectively allow variability to be more prominent.

The relationship between applied volume, moisture temperature, and wetness perception is likely a product of multimodal transduction in A-type somatosensory afferents, which account for cold cutaneous sensations and are strongly linked to the perception of wetness (25). Their collective response may be exacerbated by application of larger volumes in the preparation of stimuli. This may be caused by several factors, such as there being a larger concentration of moisture retained in the stimulus such that a higher proportion of receptors in a given area can be triggered in a form of spatial summation (27). It was also considered that changed thermodynamics at larger applied volumes may result in more stable temperatures and hence alter the physical temperature at the point of interaction, but this difference between prepared temperature and temperature at the point of contact was accounted for using data from initial sample classification studies, as noted in the methodology.

Interestingly, magnitude data have also shown positive wetness responses in dry conditions, with the neutral and warm values of 33°C and 37°C predicting low perceived wetness and the cooler values of 29°C and 25°C resulting in slightly higher wetness perceptions. Although this variation in wetness perception across different temperatures is largely consistent with data in which liquid is physically applied, it is interesting that the same principles can be applied in the absence of physical wetness such that cooler temperatures still illicit greater wetness sensations. Although existing studies have shown this influence of cold thermal inputs on wetness perception (25), the fact that there is still a positive response at the warmer temperatures despite the absence of physical wetness affirms that there are more factors involved in wetness perception. The importance of thermal cues is also reflected in the slightly different linear regression gradient associated with perceived wetness and applied volume at 33°C. As neutrality effectively lacks any form of thermal cues, neither cooling nor warming, this is likely to have caused an uncertainty in judgement which resulted in a steeper gradient compared to other temperatures.

Overall, at all applied moisture temperatures, stimuli were perceived as drier postcontact. As colder sensations are associated with an increase in wetness perception, it could be expected that wetness perceptions would actually have increased as a result of the finger being lifted and temperatures reducing postcontact. This would have been more prominent at the neutral and warm temperatures of 33°C and 37°C, respectively, as the higher temperatures would result in the participant experiencing a greater thermal gradient toward ambient, in addition to evaporative cooling. The temperatures below neutral, 25°C and 29°C, would have also been subject to some evaporative cooling, but the skin would also have increased in temperature towards neutral when in the ambient air and no longer in contact with the stimulus, effectively negating the change. Despite this, each applied moisture temperature was perceived to be drier postcontact. Although this interpretation of dryness postcontact is factually correct, it was thought that the associated evaporative cooling cues would effectively counteract or even override the dryness experienced, especially at temperatures above neutral. The fact that it does not implies that additional cues beyond thermal inputs are also involved in the sensory feedback mechanism.

### Magnitude Estimation of Thermal Perception

The effect of cooling cues on wetness perception can be further investigated by observing the corresponding thermal perceptions and physical temperature data surrounding interactions. In during- and postcontact interactions, applied volume and applied moisture temperature were found to have a significant effect on thermal perceptions. As can be expected this effect is mainly attributed to the applied temperature, but interestingly the interactive effect of applied temperature and volume was greater than the effect of volume alone. This may result from changed thermodynamics at larger applied volumes, coupled with the greater proportion of receptors being triggered in a given skin surface area as stimuli concentration increases, resulting in spatial summation and a heightened collective response. At a neutral temperature there is very little change in thermal perception across the volumes, which can be expected as there is effectively no directional change in thermal sensation that can be exacerbated by volume. This gives further evidence towards aforementioned linear regression gradients between wetness perception and applied volume, at which 33°C was steeper than other temperatures, as this is resulting from thermal sensations. Interestingly, the limited difference in thermal perception between 25°C and 29°C may account for the lack of significant differences between the absolute wetness detection thresholds at those temperatures, which is also reflected in wetness perception magnitude data at all volumes between these temperatures. This gives further evidence to the inherent link between thermal sensation and wetness perception, which is exacerbated by volume.

After expressing all thermal perception data as a deviation from the midpoint of the scale, the proportional influence of applied temperature, applied volume and their interaction shifted. As the basis for this data transformation was to align the direction of perceptual magnitudes changes and negate cancelling from their opposing directions, it could be easily predicted that the influence of volume would increase in relation to other variables. However, there is little to justify such a dramatic decrease in the influence of applied temperature. As physical surface area and liquid volume share a positive relationship with both physical temperature and thermal perceptions, both of which affect wetness perception, it could be proposed that all three aspects may be interlinked to produce such change. This is a small insight to the potential network which may underpin wetness sensations in different environmental conditions and circumstances, which should be further investigated.

The magnitude estimation data also showed a significant difference in thermal perception between during- and postcontact interactions. In each case the difference in thermal perceptions between during- and postcontact interactions was proportional to the magnitude of the applied temperature from neutrality. From a thermal perspective, this is because after the finger is lifted to perform postcontact assessments its temperature will change as it equilibrates with the ambient air. This rate of change varies greatly depending on the initial temperature, as was demonstrated in thermal profiles. For example, after interaction with a cool stimulus the finger temperature would instead increase back to neutrality, and as such the two cooler temperatures of 25°C and 29°C were perceived as warmer postcontact, effectively overcoming any influence of evaporative cooling. Conversely, after interaction with a warm stimulus the finger temperature begins to decrease back to the point of neutrality, which was shown in the two warmer stimuli of 33°C and 37°C being perceived as cooler.

It should be noted that all stimuli were perceived as drier postcontact despite thermal perceptions being varied in directionality. As thermal inputs have been shown in a range of research to have a large influence on wetness perceptions, a concept that was reflected in the wetness detection threshold data, it could be expected that the associated thermal perceptions would have had a greater influence in this case. Although the stimuli were correctly perceived as being drier postcontact, the evidence from thermal perceptions again implies that additional cues are involved in the sensory feedback mechanism and add further complexity to the processing and interpretation of wetness perception.

### Multiple Regression Analysis

Physical and perceptual factors both contributed to a statistically significant relationship within a linear regression model, accounting for 85.5% of total variance in during-contact wetness perceptions. This is to be expected, as temperature and volume form an integral part of the physical sensations associated with wetness perceptions, as previously discussed. From the linear regression equation, it can be seen that volume has a greater effect than temperature. This can be expected on a fundamental level, as larger applied volumes typically result in a greater surface area such that a larger quantity of mechanoreceptors and thermoreceptors in the skin will be activated. However, in this case all surface areas resulting from the application of moisture were sufficient to cover the average index fingerpad in females, which typically ranges from 78 mm^2^ at rest to 120 mm^2^ under low pressure (18).

Instead, the main variation caused by the application of different volumes will be the quantity of applied liquid retained in the topsheet. This is effectively the concentration of moisture contained within a given area, termed the surface wetness, and increases with higher volumes. Those stimuli with higher surface wetness will have a greater number of contact points with the skin and therefore there is a higher likelihood of activating thermoreceptors or even mechanoreceptors in the skin, resulting in spatial summation (28). Therefore, the increase in liquid volume giving rise to increased thermal stimulation may in turn effect the perceived level of wetness, which highlights an interactive effect between them. This greater level of liquid will also result in a slower thermal loss due to a lower surface area to volume ratio and can alter thermodynamics such that there is decreased thermal conductance at larger applied volumes. Conversely, if only a relatively small quantity of liquid is transferred to the finger from the stimuli such that a thin liquid layer is present on the skin, it is more susceptible to evaporative cooling (29).

Although interlinked with volume, temperature is also a significant part of the wetness detection process in its own right. This is shown simply by the wetness perception during-contact increasing as temperatures lower, across all applied volumes. This is also presented in existing literature, such as research by Filingeri et al. (25) showing that cold wet stimuli were perceived as significantly wetter than neutral wet and warm wet stimuli of the same volumes. This again shows that cold thermal afferents are of primary importance in underpinning the perception and flexibility of skin wetness perception during-contact with an external stimulus and may form the basis of a larger multisensory model.

### Conclusions

Using a dichotomous response method, the wetness detection threshold of the human index finger at the moisture temperature resembling human skin temperature (i.e., 33°C) was 24.7 ± 3.48 mL. This threshold could be modulated according to applied moisture temperature, with mean wetness detection thresholds varying by 8.3 mL between the lowest and highest applied moisture temperatures of 25°C and 37°C. Overall, wetness detection thresholds were lower at cooler temperatures and higher at warmer temperatures. This forms a positive relationship and indicates that lower temperatures result in greater wetness sensations. The directionality of relationship was analogously observed in magnitude estimation data, a method which also highlighted the difference between during- and postcontact interactions. This research has identified and quantified several factors contributing to the network of human wetness perception. It can be used as a foundation to form a predictive model for integrating other sensory modalities involved in wetness sensation and assessing additive, synergistic, or antagonistic components of their interactions. The resulting insights will inform the design of future academic studies and aid the development of superabsorbent hygiene products, enhancing their comfort, economy, and effectiveness.

## GRANTS

The present research was conducted in the context of an industry co-funded Ph.D. from Loughborough University; The Engineering and Physical Sciences Research Council and Procter and Gamble Service GmbH provided financial support.

## DISCLOSURES

No conflicts of interest, financial or otherwise, are declared by the authors.

## AUTHOR CONTRIBUTIONS

C.M., R.R., and D.F. conceived and designed research; C.M. performed experiments; C.M. and D.F. analyzed data; C.M., R.R., and D.F. interpreted results of experiments; C.M. prepared figures; C.M. drafted manuscript; C.M., R.R., and D.F. edited and revised manuscript; C.M., R.R., and D.F. approved final version of manuscript.
